# Remembering Johann Gregor Mendel: a human, a Catholic priest, an Augustinian monk, and abbot

**DOI:** 10.1002/mgg3.186

**Published:** 2015-11-11

**Authors:** Father Clemens Richter

**Affiliations:** ^1^ OstfildernGermany

## Abstract

Johann Mendel (Gregor was the name given to him only later by his Augustinian order, Fig. 1) was born on July 20, 1822 to an ethnic German family, Anton and Rosina Mendel (Fig. 2), in Heinzendorf in the Austrian Empire at the Moravian‐Silesian border (now Hynčice, Czech Republic).

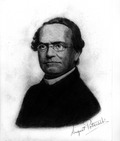

In the house of my grandparents in Heinzendorf (today called Vranze), there was a room that was only opened on special occasions and entered with utmost respect. In it was a framed picture of an ecclesiastical dignitary dressed in a violet‐red gown with white, magnificently embroidered rochet carrying a precious crucifix on his chest. Only much later did I learn that this person was my great‐great‐granduncle: the prelate Johann Gregor Mendel, the abbot of the Augustinian Abbey St. Thomas of Alt Brünn (Old Brno), and the discoverer of the laws of inheritance. As an Augustinian monk myself, I feel very close to my famous relative. Please allow me to remember him as a human being and a Catholic priest as well.

Johann Mendel (Gregor was the name given to him only later by his Augustinian order, Fig. 1) was born on 20 July 1822 to an ethnic German family, Anton and Rosina Mendel (Fig. 2), in Heinzendorf in the Austrian Empire at the Moravian‐Silesian border (now Hynčice, Czech Republic). The Christian spirit, present in this deeply religious peasant family, is well documented by a little burned tile found in the Mendel living room. On it was the Holy Trinity symbolized by three intertwined circles that include the words: “Dein Wille geschehe.” (“Thy will be done.”) These three words may well have been an imprint on young Mendel and his two sisters, Veronika and Theresia. Here, the roots were laid for his later wish to become a priest.

**Figure 1:**
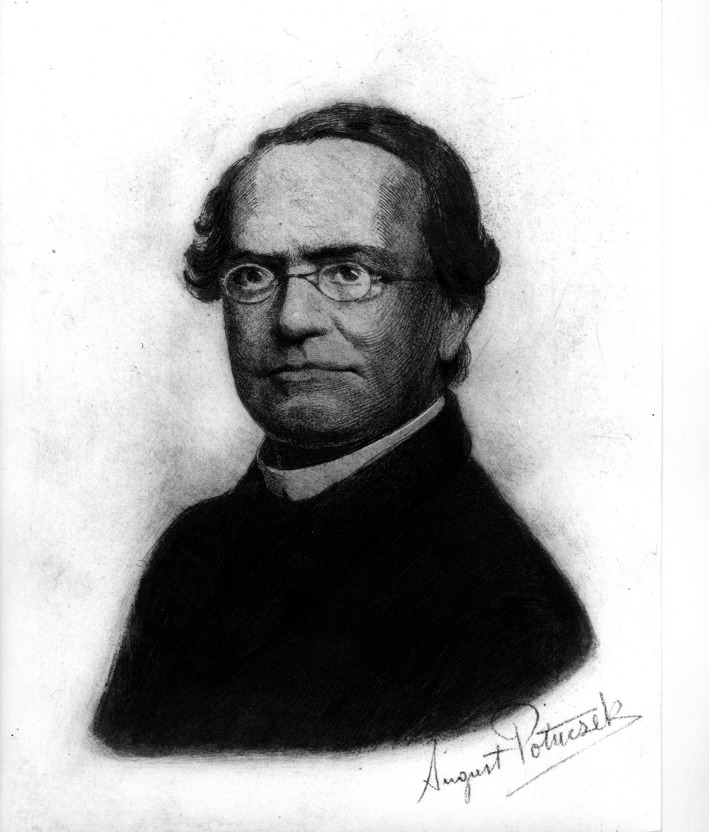
Drawing of Johann Gregor Mendel (1822 to 6 January 1884) by August Potuczek.

**Figure 2:**
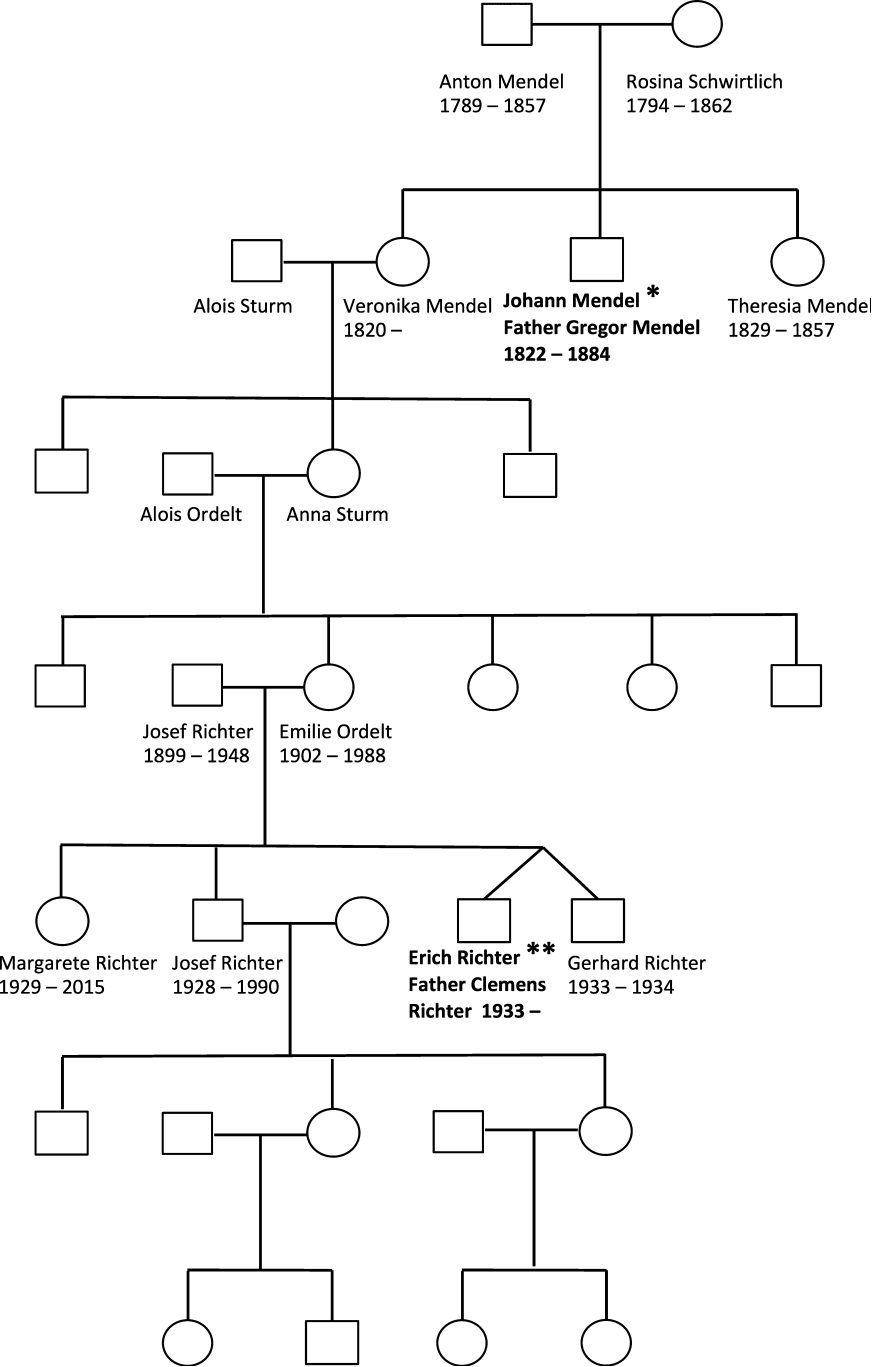
Pedigree of Johann Gregor Mendel (_*_) and his great‐great‐grandnephew Father Clemens Richter (_**_), both Augustinian monks.

In 1843, Johann Mendel was admitted as a novice to the Augustine Monastery in Brünn (now Brno, Czech Republic) by Abbot Cyrill Napp, the same abbot that Gregor Mendel would eventually succeed. On 25 December 1846, Mendel became an ordained priest and this documentation is still preserved in the museum next to the monastery. All through his life Mendel whole‐heartedly assented to his vows; he truly accepted all obligations laid onto him by his order. How else would his fellow‐brethren have elected him abbot unanimously in 1866 after the death of Cyrill Napp? Mendel was deeply rooted in his Christian faith, and he passionately tried to convey his conviction and experience to others at any given occasion. Testimony of this attitude is shown in various outlines of sermons that are still preserved.

In stature, Mendel was medium sized, with broad shoulders and a bit of a stout figure. He was well liked by others, always ready to talk. He had a good sense of humor and of “comedy of a situation.” His contemporaries emphasized his winning character. One of his fellow monks spoke of Mendel as “affabilis unicuique” (kind and friendly to everyone). “By his generosity, kindness and mild‐manneredness, Mendel acquired universal respect and sympathy. No appeal for help he left unanswered and, in an amicable manner, he knew how to dispense help without letting the petitioner feel the charity.” (Quotes from one of his obituaries).

Certainly, Mendel's honest character, his great merits in church, school, and science as well as his support of national tolerance held him in high esteem. As the Abbot, Mendel accepted more Czech novices than German applicants – overseeing the transition of a mainly German‐speaking to a Czech‐speaking monastery. Mendel's idea for the coexistence of different nations in the microcosm of St. Thomas Abbey was the reverence for his fellow human beings and their diversity and the mutual responsibility of members of both nations for the cultural and economical development of their common homeland. Today, we would consider Mendel a true European or world citizen.

In Mendel's life, his last decade was overshadowed by a hopeless conflict. As the Abbot of the monastery, Mendel had to fight with the Austrian government over an exorbitant taxation of his abbey. With all his resoluteness he refused the payment from the first day of the new law until his death. This exhausting fight darkened the last years of his life; he felt misunderstood, even by his own fellow monks who discouraged him to proceed. Only a few years after his death did the Austrian government withdraw the taxation of his and other monasteries. They even paid back some of the previously received taxes. In a sermon given by Mendel during his latter years he revealed how seriously he suffered from such injustices: “How strange it may seem to a faithful Christian to be told of victory amidst this unrighteous world rather than to hear again of disregard, defamation, and prosecution.” With such words he certainly did not only reflect on the unjust treatment of Christ in his Passion, but also the injustice to which he was exposed. But continuing with his sermon, he then showed how to avoid bitterness and that all injustice in worldly affairs is overcome by the eternal glory and omnipotence of God. Similarly to his futile fight against the Austrian government, he may have viewed his scientific achievements unappreciated by his contemporaries: Mendel considered the results and impact gained from his *Experiments in Plant Hybrids* as fundamental. However, they were not understood and acknowledged during his lifetime. A few months before his death he told a novice in the monastery, Franz Barina who later became his successor as abbot: “Even though I have experienced some dark hours during my life time, I am grateful that the beautiful hours have outweighed the dark ones by far. My scientific work has brought me great joy and satisfaction; and I am convinced that it won't take long that the entire world will appreciate the results and meaning of my work.” To a friend he expressed his firm opinion: “Meine Zeit wird kommen” (my time will come).

Hugo Iltis, the first biographer of Mendel writes, “Mendel's thinking was geared towards the concrete, reflections and sentimentalities were not of it.” He never wrote a diary; 59 letters written to family members and friends do not elucidate his inner life. Toward the end of his life, he became more and more lonely. “When he died,” writes Hugo Iltis, “no one was aware of the importance of Mendel's work, and the few hand‐written notes were carelessly put aside or burnt.”

At the centennial celebration in 1965 in Brünn, it was said that “Mendel whose language is that of a Luther, Leibnitz, Kant, and Goethe, today not only belongs to the international scientific world, but he belongs to all of us.” Mendel was a gifted and ingenious researcher, a noble human being, a faithful Christian, a good priest, and a devoted son of his homeland.

